# Engineered Bacteriophages: A Next-Generation Platform for Precision Antimicrobials and Therapeutics

**DOI:** 10.3390/v18030355

**Published:** 2026-03-13

**Authors:** Haonan Shao, Youpeng Deng, Yongpeng Shi, Yi Duan

**Affiliations:** 1 State Key Laboratory of Immune Response and Immunotherapy, Department of Infectious Diseases, The First Affiliated Hospital of USTC, Center for Advanced Interdisciplinary Science and Biomedicine of IHM, Division of Life Sciences and Medicine, University of Science and Technology of China, Hefei 230026, China; haonan_shao@mail.ustc.edu.cn (H.S.); youpengd@mail.ustc.edu.cn (Y.D.); shiyongpeng@ustc.edu.cn (Y.S.); 2 Key Laboratory of Anhui Province for Emerging and Reemerging Infectious Diseases, Hefei 230027, China

**Keywords:** bacteriophage, phage therapy, engineered phages, precision medicine, synthetic biology

## Abstract

The escalating crisis of antimicrobial resistance (AMR) and the stagnating antibiotic pipeline have renewed interest in bacteriophage therapy. While natural phages offer specificity and low toxicity, their narrow host range, bacterial resistance, and safety concerns limit clinical use. To overcome these hurdles, phages are being engineered using biotechnology. This review outlines the history of phage therapy and systematically summarizes advances in engineered phage preparation, including genetic modification, chemical conjugation, and physical encapsulation. We highlight the application of engineered phages against multidrug-resistant infections, gastrointestinal diseases through gut microbiome modulation, and as targeted delivery vehicles or immune adjuvants in cancer therapy. While significant advances have been made, several critical challenges remain, particularly in regulatory approval, large-scale manufacturing, and ensuring long-term safety. We conclude that engineered phages, as customizable and precise biological tools, are poised to advance precision phage medicine, offering a transformative solution to AMR and fostering convergence across synthetic biology, medicine, and environmental science.

## 1. Introduction

The discovery and application of antibiotics represent one of the most significant milestones in the history of modern medicine. They facilitated the safe performance of complex surgical procedures, transformed the treatment of bacterial infections, and significantly reduced global mortality from infectious diseases [1]. However, in recent years, the escalating global crisis of bacterial antibiotic resistance coupled with the declining pipeline of novel antibiotic development has necessitated the exploration of alternative therapeutic strategies [2,3,4]. In this context, bacteriophages (phages), naturally occurring viruses capable of specifically infecting bacteria, have regained significant attention as a promising alternative.

As ubiquitous natural antimicrobial entities [5,6], phages can specifically infect their host bacteria, hijack the bacterial replication machinery for propagation, and co-evolve with their hosts, all while exhibiting low inherent toxicity to humans. These attributes make phage therapy a potentially powerful approach to combat antibiotic-resistant infections [7,8,9], holding considerable promise for future clinical applications and several recent cases have demonstrated the successful use of phages in treating bacterial infections [10,11,12]. Nevertheless, in spite of the vast therapeutic potential embedded within natural phage libraries, their clinical translation faces multiple challenges, including narrow host ranges [13], the ease with which bacteria develop phage resistance [14], undefined immunogenicity [15], and other potential risks.

To overcome these limitations and maximize the antibacterial potential of phages, researchers have integrated high-throughput sequencing, metagenomics, genetic engineering, synthetic biology, and molecular biology techniques to develop engineered phage therapies [16,17,18,19,20]. These approaches enable the introduction of novel functions and properties that transcend natural evolutionary constraints. To facilitate further research in this field, this review outlines the development of phage therapy, systematically summarizes recent advances in engineered phage approaches, and discusses current limitations along with proposed future directions.

## 2. Phage-Based Therapy: History and Limitations

The bactericidal activity of phages was first documented in 1896 [21], but it was not until 1917 that Felix d’Herelle coined the term “bacteriophage” [22]. In 1919, he conducted the first documented phage therapy, successfully treating shigellosis using a *Shigella* phage [8]. Subsequent studies worldwide confirmed the therapeutic potential of phages against bacterial infections [23,24,25]. However, scientific interest in phage therapy declined with the industrialization of conventional antibiotics [26]. Recent decades have witnessed a resurgence of interest due to the escalating challenge of antimicrobial resistance [27,28,29], positioning phage therapy as a promising alternative [30,31,32,33,34,35].

Although phage therapy has a long history and has renewed interest, it is crucial to recognize that phage therapy still faces inherent biological and technical challenges. For instance, the high host specificity of phages, while advantageous for targeted effect, inherently restricts their host range [13,36]. This selectivity is primarily determined by phage recognition of and binding to specific bacterial surface receptors [37]. This receptor-dependent mechanism, however, allows bacteria to develop resistance through receptor mutations. To extend the therapeutic lifespan and broaden the spectrum, conventional approaches often employ phage cocktails—mixtures of multiple phages targeting diverse receptors. Nevertheless, the development of such cocktails is a complex and time-consuming process involved in their manufacture and the tracking of their pharmacodynamic and immunogenic properties, which may hinder their advancement as antimicrobial agents and the timeliness of clinical application [38]. Besides, the long-term co-evolution between phages and bacteria renders pathogenic bacteria prone to developing resistance during phage therapy [14,39,40]. In addition, safety concern presents another critical challenge. As foreign biological entities, phages may harbor and express virulence genes, potentially causing adverse effects in humans, and can interact with the host immune system, triggering unintended responses [15,41,42,43,44]. Furthermore, most therapeutic phages are lytic [45], and their bacteriolytic activity may disrupt the stability of the gut microbiota and metabolome [46]. Compounding these challenges is our still-limited understanding of phage functional genomics and proteomics. At the technical level, the long-term stable preservation and activity maintenance of bacteriophages remain a critical bottleneck in their development and clinical translation. Although current methods such as cryopreservation and lyophilization enable short-term storage, including activity decay, suboptimal formulation compatibility, high environmental sensitivity, and a lack of standardized quality control protocols. Collectively, these factors hinder the broader application of conventional phage therapy.

## 3. Engineered Phages

Synthetic biology advancements and artificial intelligence-driven algorithmic models enable phages to transcend the constraints of natural evolution, driving their development toward broader-spectrum, more efficient, and safer engineered customization. Natural phages typically exhibit narrow host ranges, making it difficult to identify suitable combinations for conventional therapy. Reprogramming phage host specificity through genetic modification can overcome this limitation. Engineered phages can be designed to alter or broaden host tropism through high-throughput and targeted approaches, such as modifying host recognition proteins or editing genes determining specificity [47,48]. For example, site-directed mutagenesis of the host range determinant region (HRDR) in the tail fiber protein of phage T3 has been used to expand its host range and construct engineered phage libraries [49]. Bacteria have evolved diverse anti-phage defense mechanisms throughout their long co-evolutionary arms race with phages. Wild-type phages have low natural mutation rates, making it difficult to obtain desirable mutants through directed evolution within short time frames. Many of these bacterial resistance mechanisms, however, can be circumvented using engineered phages. For instance, numerous bacteria evade phage predation by altering cell wall-associated receptors. In such cases, phage host range can be broadened through targeted engineering of receptor-binding proteins (RBPs) [47,50]. To enhance the infectivity and lytic efficiency of phages against resistant bacteria, gene editing techniques can be employed to modify phage genomes [42]. For example, overexpression of phage-encoded lytic proteins can strengthen the bacteriolytic activity of engineered phages [51,52]. Bacterial immune mechanisms such as CRISPR-Cas systems can be subverted by introducing anti-CRISPR (acr) genes into engineered phage genomes [53,54]. Additionally, many phages carry virulence or toxin genes, rendering them unsuitable for therapeutic use. This critical safety concern can be mitigated by precisely removing potentially harmful genetic elements from the viral genome [55]. Genome reduction--by knocking out non-essential genes--not only enhances controllability and safety but also creates space for introducing foreign genetic elements [56]. Engineered phages can serve as targeted delivery vehicles to transport gene-editing systems into bacteria, enabling in situ modulation of bacterial gene expression without compromising viability [57]. Moreover, toxin release resulting from phage-induced lysis may cause severe side effects during therapy. To address this, non-lytic phage variants have been developed. For example, a recombinant *Staphylococcus aureus* (*S. aureus*) phage deficient in endolysin was constructed that does not lyse bacterial cells but remains lethal against methicillin-resistant *S. aureus* (MRSA) [58]. This modified phage demonstrated high efficacy in rescuing mice infected with a lethal dose of *S. aureus*. Similarly, lysin-deficient engineered phages targeting *Pseudomonas aeruginosa* (*P. aeruginosa*) or *Escherichia coli* (*E. coli*) provided better protection in mice challenged with lethal bacterial doses compared to wild-type phages [59,60]. Finally, phage engineering also offers a powerful platform for exploring the functions of unknown proteins [49].

**Figure 1:**
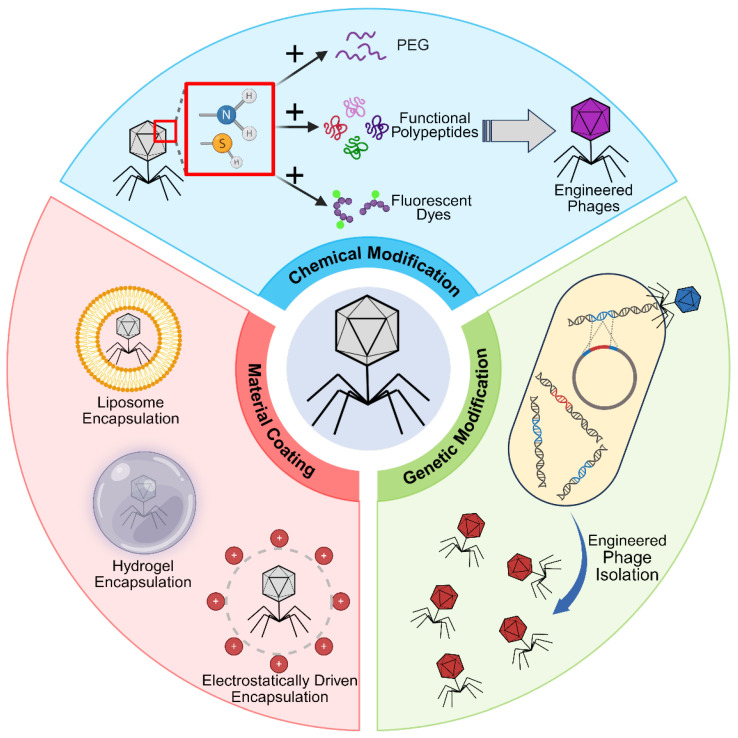
Schematic illustration of engineering strategies of phage. The engineering strategies for bacteriophages can be broadly categorized into three forms: genetic modification, chemical modification, and material coating. Genetic engineering typically involves the insertion of a gene editing system from a pre-constructed engineered plasmid in a production strain into the bacteriophage genome via homologous recombination, resulting in engineered phages. Chemical modification generally utilizes the abundant reactive groups on the surface of phage capsid proteins to anchor exogenous functional molecules through chemical conjugation. Material coating utilizes non-covalent physical interactions to assemble phages with functional nanomaterials to construct multifunctional nanocomplexes.

As previously discussed, engineered phages exhibit significant advantages in targeting specificity, bactericidal efficacy, and functional synergy by overcoming the limitations of their natural forms. However, these superior properties are not achieved arbitrarily—they rely fundamentally on a series of sophisticated preparation technologies. Currently, these techniques are broadly categorized into three main strategies: genetic modification, chemical modification and material coating (Figure 1). This section elaborates on the principles, methodologies, and recent advances of these key technologies, aiming to illuminate the technical pathway through which engineered phages transition from concept to implementation.

### 3.1. Genetic Modification

The most fundamental and enduring strategy involves conferring or enhancing specific functions at the level of the genetic blueprint through precise insertion, deletion, replacement, or regulation of the phage genome. Phage genetic engineering commonly employs homologous recombination [61], which exploits the native DNA repair machinery of host cells. By introducing an exogenous DNA fragment carrying desired mutations with high homology to the target sequence, the cellular machinery is “deceived” into integrating the foreign fragment into the phage genome, thereby achieving genetic editing. However, this method suffers from inherently low recombination efficiency and high randomness, often requiring extensive screening to obtain progeny phages with the desired mutations [20,62]. Fortunately, incorporating recombination proteins (e.g., the phage-derived λ Red or Rac RecE/RecT systems) into the host significantly enhances recombination success. These systems protect editing templates from degradation and facilitate their annealing with the injected phage genome (in vivo recombineering), thereby increasing recombination frequency and reducing the required length of homologous arms [20]. To address the laborious and time-consuming screening process, researchers have integrated positive and negative selection mechanisms. For instance, reporter genes (e.g., luciferase or fluorescent proteins) or phage-specific marker genes (e.g., *trxA* or *cmk* in coliphages) are inserted during homologous recombination, substantially improving the selection efficiency of engineered phages [63,64]. Encouragingly, the development of CRISPR-Cas counter-selection methods has further streamlined the screening process [51,65]. Following recombination, the CRISPR-Cas system within the host bacterium can selectively cleave unedited phage DNA, leading to the death of wild-type phages and enrichment of the engineered variants [66].

Synthetic biology also plays a pivotal role in the construction of engineered phages. It involves rational design, de novo synthesis, and transformation tools to significantly enhance phage infection efficiency and overcome limitations associated with natural phages [67,68]. Utilizing synthetic biology approaches for phage genome engineering has enabled the design of phage genomes capable of infecting and killing a broad spectrum of bacterial species [67,68,69]. Moreover, synthetic biology can be synergistically combined with reboot technology [47]: synthetic biology provides the “design concepts” and “construction methods” that determine the functions of the phage, while reboot technology offers the “activation means” and “production platform” that determine whether and how the designed genome can be “revived” into viable phages [70,71]. Together, they form a complementary and closed loop from design to functional production.

### 3.2. Chemical Modification

This strategy involves directly anchoring exogenous functional molecules onto the phage capsid surface via chemical conjugation reactions utilizing reactive groups present on capsid proteins, without altering the genetic material. It represents a relatively rapid and versatile functionalization approach. The abundant functional groups (e.g., amino, hydroxyl, thiol groups) on the phage capsid provide reactive sites for specific conjugation [72]. Chemical modification can endow phages with novel functionalities, such as enhanced targeting, improved pharmacokinetics, and theranostic capabilities [73,74]. Importantly, moderate chemical modification of capsid proteins does not significantly compromise the structural integrity of phage particles or their inherent infectivity [75], allowing the addition of various functional molecules to the surface without interfering with their biological functions. There are some commonly applied functional modifications:

For instance, modifying phage endolysins with cationic peptides significantly enhances their ability to penetrate the cell membranes of Gram-negative bacteria, enabling efficient antibacterial activity independently of membrane-permeabilizing agents [76].

Beyond therapeutic enhancement, this approach is highly valuable for diagnostic purposes. Conjugating fluorescent dyes, contrast agents (e.g., for MRI or CT), or reporter molecules to phages allows real-time imaging and localization of targets (e.g., tumors or infection sites) [77,78].

When the goal is to improve in vivo performance for delivery or therapy, attaching polymers such as polyethylene glycol (PEG) to phages confers “stealth” properties, reducing recognition and clearance by the host immune system, prolonging circulation time, and improving delivery to and action at the target site [79].

Moreover, engineering phage-displayed peptide libraries via chemical cyclization strategies (e.g., N-terminal cysteine-mediated cyclization) facilitates the selection of cyclic peptides with superior stability, affinity, and membrane permeability. These cyclic peptides are valuable resources for developing protein–protein interaction inhibitors or drug lead compounds [75,80].

### 3.3. Material Coating

Material Coating involves the physical assembly of phages with functional nanomaterials via non-covalent interactions (e.g., electrostatic, avidin-biotin, antibody-antigen) to construct multifunctional “nanohybrids” that largely preserve the original functions of each component. Phage therapy is typically administered orally, topically, or via injection [81]. Before reaching their target, phages must endure environmental challenges such as gastric acid degradation, systemic clearance, and cellular membrane barriers [82], all of which may impair their stability and efficacy prior to encountering the target bacteria. Material coating offers an effective solution to these challenges. Based on the coating materials and strategies employed, encapsulation can be broadly classified into the following categories:

A widely used method is liposome Encapsulation, in which phage aqueous solutions are mixed with phospholipid materials using thin-film hydration or reverse-phase evaporation methods to form liposomes encapsulating phages under appropriate conditions. By adjusting phospholipid composition, cholesterol ratio, and surface modifications (e.g., PEGylation), the stability, encapsulation efficiency, and targeting of liposomes can be optimized. Liposomes provide excellent protection by shielding phages from gastric acid (low pH) and bile salt damage, as well as immune system attacks [83,84,85,86,87]. Moreover, the cell membrane-like structure of liposomes confers high affinity for epithelial cell membranes, enhancing adhesion to intestinal cells. The positive charge of cationic liposomes interacts with negatively charged components in the mucus layer (e.g., sialic acid and sulfated mucins), further improving mucoadhesion [88].

Beyond lipid-based systems, hydrogel encapsulation offers another robust platform, utilizing biodegradable materials (e.g., PLGA, sodium alginate, chitosan) to provide physical isolation and protection. The polymer matrix offers a robust physical barrier, exhibiting strong resistance to gastrointestinal environments [89,90]. The high-water-content environment of hydrogels and the biocompatibility of natural polymers create a favorable microenvironment for phages. Additionally, controlled release can be achieved by tuning polymer type and cross-linking density [91].

In contrast to the matrix-based methods above, electrostatically driven encapsulation relies on direct interactions between the negatively charged phage surface and cationic materials (e.g., DOTAP). Many phages carry a net negative charge on their capsid surface at physiological pH. Mixing them with cationic polymers (e.g., chitosan, poly-L-lysine) or cationic liposomes enables the formation of complexes or core–shell structures via electrostatic adsorption, resulting in encapsulation [92]. This method is generally simple, rapid, and does not require complex equipment or harsh reaction conditions. Studies have shown that electrostatic-based encapsulation strategies can achieve high encapsulation efficiency (>90%) with low phage titer loss (<0.2 log_10_) [93].

Advancing toward more sophisticated release control, stimuli-responsive encapsulation employs coating materials designed to respond to specific gut environmental cues (e.g., pH [90,94], enzymes [95,96,97]), enabling dissolution or degradation at targeted sites. This approach achieves high levels of targeting and precise release, significantly improving the efficiency of phage-mediated gut microbiota editing and reducing the required dosage.

## 4. Engineered Phages and Human Diseases

### 4.1. Engineered Phages in Multidrug-Resistant Bacterial Infections

As mentioned earlier, the misuse of antibiotics has led to the global spread of multidrug-resistant (MDR) bacteria, renewing interest in phage-based therapeutics. Although natural phages demonstrate considerable efficacy in clearing infections, their repeated use may lead to phage resistance [98]. Engineered phages, however, have shown promise in addressing challenges faced by both natural phages and conventional antibiotics in combating MDR and extensively drug-resistant (XDR) bacteria [99,100] (Table 1).

Dedrick et al. reported the first clinical use of a genetically engineered phage cocktail, successfully treating a disseminated *Mycobacterium abscessus* (*M. abscessus*) infection in a cystic fibrosis patient, thereby demonstrating the feasibility of personalized phage therapy [42]. To enhance efficacy, Qin et al. constructed engineered phages with anti-CRISPR genes (EATPs), which exhibited potent antibacterial activity against MDR *P. aeruginosa* and reduced associated antibiotic resistance [101]. Similarly, Nick et al. genetically modified a mycobacteriophage by deleting a lysogeny repressor gene, enhancing its lytic capability against *M. abscessus* and achieving successful therapeutic outcomes [102]. Encouragingly, Zhao et al. developed the CRISPR-Cas9-engineered phage selz_HA-TAT_, which is capable of traversing mammalian cells to clear intracellular bacteria, thereby effectively extending the scope of phage therapy to intracellular pathogens [103]. To address the challenge of phage resistance, Yehl et al. identified HRDRs in phage tail fibers and employed directed evolution to broaden host targeting and suppress resistance emergence [49]. Wang et al. further advanced the field by creating a synthetic phage library using a universal safe chassis and well-characterized receptor-binding proteins, enabling predictable host targeting and streamlined development [104]. Selle et al. armed a phage with a modified CRISPR-Cas3 system, achieving specific depletion of *Clostridioides difficile* (*C. difficile*) in vivo without disrupting the gut microbiota [105]. Dong et al. developed an innovative biohybrid platform combining engineered phages with ROS-responsive nanoparticles for controlled polymyxin B release, circumventing resistance and improving survival in a murine model of Gram-negative bacterial pneumonia [106]. Bikard et al. employed an engineered *Staphylococcus aureus* phage delivering CRISPR-Cas to selectively eliminate antibiotic-resistant strains, suppress virulence genes, and prevent resistance through multiplexed targeting [107]. Beyond whole phages, engineered phage enzymes (EPEs) have shown promise. Defraine et al. utilized the Artilysin Art-175 to effectively kill MDR *Acinetobacter baumannii* (*A. baumannii*), including polymyxin-resistant strains [108].

### 4.2. Engineered Phages in Gastrointestinal Diseases

Engineered phages show great potential not only in systemic MDR infections but also in treating gut-related disorders (Table 2). The intestine is a unique ecosystem characterized by extreme physicochemical conditions (e.g., low gastric pH, bile salts, digestive enzymes), a dense microbial community (gut microbiota), and a localized immune system. It is also an ideal arena for engineered phages to exert precise regulatory functions.

Alterations in the human gut microbiome are closely linked to gastrointestinal and hepatic diseases, including inflammatory bowel disease (IBD), colorectal cancer (CRC), alcoholic liver disease, and non-alcoholic fatty liver disease (NAFLD) [23]. Through genetic engineering, chemical modification, and especially physical encapsulation, engineered phages can achieve colon-targeted delivery, precisely eliminate pathogens (e.g., *C. difficile*, antibiotic-resistant *E. coli*), and even modulate microbiota composition, neutralize toxins, and regulate local immune responses. This offers new perspectives for treating non-infectious intestinal diseases such as IBD, NAFLD, and colitis.

As an example, Baker ZR et al. utilized the coexistence of phages with their bacterial hosts to generate a sustained in situ effect, orchestrating the production and release of the heterologous protein Serpin B1a in the mammalian gut [109]. This approach reduced neutrophil elastase (NE) activity in the mouse intestine during colitis and effectively alleviated colitis symptoms while suppressing obesity development in murine models. Furthermore, engineered phages can selectively inhibit the proliferation of pathogenic bacteria while promoting the growth of beneficial microbes in the murine gut, thereby effectively restoring microbial ecological balance and enhancing intestinal barrier integrity. Zhu JY et al. engineered a lytic phage targeting ST11 KL64 carbapenem-resistant *Klebsiella pneumoniae* (CRKP) by incorporating cerium dioxide (CeO_2_) nanozymes, endowing the phage with potent antibacterial, anti-biofilm, and anti-inflammatory capabilities [110]. This modified phage significantly suppressed the transcription of host genes related to energy and amino acid metabolism, ultimately inducing bacterial death. Beyond their direct bactericidal effects, engineered phages serve as precise delivery vehicles for bacterial targeting. Brödel AK et al. combined phage-derived particles with base editors to achieve in situ, precise gene editing of specific bacteria within the mouse intestinal tract for the first time [57]. This system enables therapeutic editing of bacterial pathogenicity genes, such as those conferring antibiotic resistance or encoding toxins, offering a novel strategy for microbiome-targeted treatment of intestinal diseases.

### 4.3. Engineered Phages in Adjuvant Cancer Therapy

Conventional cancer treatments such as surgery, radiotherapy, and chemotherapy have advanced considerably but still face challenges, including recurrence, metastasis, and systemic toxicity [111,112]. In recent years, the tumor microenvironment (TME) has emerged as a new focus in anticancer therapy for its role in promoting tumor progression [113]. Within this complex milieu, bacteria have been revealed to play key roles in regulating multiple malignant processes [114,115,116,117], including immunosuppression, metastasis, therapy resistance, and metabolism. Meanwhile, phages—with their inherent precision for bacterial targets and high engineerability—are providing an unprecedented new paradigm for cancer treatment.

CRC patients exhibit elevated levels of the tumor-promoting bacterium *Fusobacterium nucleatum* (*F. nucleatum*) in their fecal microbiota, alongside a reduction in anti-tumorigenic butyrate-producing bacteria such as *Clostridium butyricum* (*C. butyricum*). *F. nucleatum* accumulates in tumor tissues and induces autophagy in CRC cells via the TLR4-Myd88 pathway, thereby conferring resistance to chemotherapy. In contrast, butyrate secreted by butyrate-producing bacteria like *C. butyricum* inhibits CRC cells. To suppress the over-proliferation of *F. nucleatum*, Zheng DW et al. developed a precise delivery system utilizing a phage isolated from human saliva that specifically targets *F. nucleatum* as a guidance system [118]. This system involves loading the CRC chemotherapy drug irinotecan onto dextran nanoparticles and conjugating azide-modified phages to the drug-loaded nanoparticles via covalent bonds, forming a phage-guided nanomedicine. This approach significantly reduced *F. nucleatum* levels, offering novel insights and strategies for enhancing the efficacy of colorectal cancer treatment.

Beyond directly eliminating pro-carcinogenic bacteria to suppress tumor growth, phages can also act as immune adjuvants, further enhancing antitumor efficacy. Dong X et al. constructed a bioinorganic hybrid system combining M13 phages with silver nanodots, which precisely targets and modulates *F. nucleatum* in the gut [119]. This system further facilitates the lethal effect of AgNPs on *F. nucleatum* at the tumor site. By inhibiting the proliferation of *F. nucleatum*, the recruitment of immunosuppressive cells in the colorectal tumor microenvironment can be effectively suppressed, thereby improving the gut microecology. Simultaneously, M13 phage, as a virus, possesses capsid proteins that can effectively elicit immune responses in the host, significantly activating antigen-presenting cells and further reversing the immunosuppressive microenvironment.

To enhance the specificity of cancer drug therapy, Li Y et al. pioneered the use of genetically engineered phages for targeted inhibition of tumor angiogenesis in breast cancer therapy [120]. Although conventional anti-angiogenic drugs can inhibit tumor vascularization, they often lack tumor specificity and tend to cause systemic side effects. Angiogenin, a protein overexpressed and secreted by tumors that triggers angiogenesis to support their growth, had not previously been exploited as an anti-angiogenic target. The researchers ingeniously displayed a homing peptide specific to breast cancer cells on the tip of filamentous fd phages, ensuring precise accumulation of the phages in tumor tissues. Additionally, they displayed numerous peptides with high affinity for angiogenin on the sidewalls of the phages, enabling effective “neutralization” of tumor-secreted angiogenin and inhibition of angiogenesis. Since phage display technology allows for the screening of homing peptides targeting different cancers, this angiogenin-binding phage platform can be regarded as a versatile system. Theoretically, by exchanging different homing peptides, it can be tailored to target various tumor cells, providing new perspectives and tools for cancer therapy.

**Table 1:** Summary of engineered phage-based therapeutic interventions in infectious diseases.

Disease	Study Year(s) and Location	Status	Population and Treatment Method	Dose and Frequency	Outcome	References
Disseminated, drug-resistant *M. abscessus* infection	2015–2019; London, UK	Clinical case	15-year-old girl with Cystic Fibrosis and a post-lung transplant disseminated *M. abscessus* infection Intravenous and topical administration	A three-phage cocktail (10^9^ PFU intravenously, every 12 h for at least 32 weeks; 10^9^ PFU topically, once daily;)	Intravenous phage treatment was well tolerated and associated with objective clinical improvement.	[42]
Multidrug-resistant *P. aerginosa* infection	2022; Sichuan, China	Preclinical	Human embryonic kidney cells (HEK293T) in vitro and C57BL/6N mice in vivo infected with clinically isolated MDR *P. aeruginosa* PA154197 Intraperitoneal injection in mice	One phage (10^8^ PFU in vitro; 1.2 × 10^9^ PFU, single intraperitoneal injection)	EATPs effectively inhibited the growth of clinically isolated MDR *P. aeruginosa* (60–80% inhibition rate in vitro). Significantly increased survival rates and attenuated tissue damage.	[101]
*M. abscessus* pulmonary infection	2020–2022; University of Pittsburgh, PA, USA	Clinical case (compassionate use)	26-year-old man with Cystic Fibrosis, advanced bronchiectasis, and a chronic, treatment-refractory *M. abscessus* pulmonary infection. Intravenous administration	A two-phage cocktail (10^9^ PFU of each phage intravenously, twice daily)	The patient’s clinical status improved sufficiently and successfully receive a lung transplant, no viable *M. abscessus* was cultured from extensive sampling of the explanted lungs.	[102]
*C. difficile* infection	2020; North Carolina, USA and Sherbrooke, Canada	Preclinical	Mouse model of *Clostridioides difficile* infection Oral administration	One phage (10^8^ PFU, twice daily for 4 days)	The CRISPR-enhanced phage (crPhage) showed significantly better killing of *C. difficile* in vitro and reduced bacterial load in mouse.	[105]
*Salmonella* infection	2023; Chongqing and Shenzhen, China	Preclinical	Multiple mammalian cell lines infected with *Salmonella Typhimurium* SL1344 In vitro treatment	N/A (in vitro)	Significant reduction in intracellular *Salmonella* counts in Hela (64%) and A549 (48%) cells	[103]
Bacterial infections	2019; Cambridge, MA, USA	Preclinical	*E. coli* strains, and a mouse skin infection model In vitro treatment and topical administration	One phage (10^9^ PFU, single topical administration)	Significantly reduced bacterial counts in a mouse skin infection model without promoting resistance and no observed bacterial resistance to phages over long-term culture.	[49]
Multidrug-resistant and carbapenem-resistant *K. pneumoniae* infections	2023; Shaanxi, China	Preclinical	238 clinical *K. pneumoniae* strains with diverse capsular types, and a library of 114 sequenced phages. In vitro treatment	N/A (in vitro)	Successfully engineered phages by RBP swapping: Shifted host tropism from KL2 to KL57, Broadened host range to simultaneously target KL1, KL2, and KL57 strains.	[104]
Multidrug-resistant Gram-negative bacterial pneumonia	2025; Chongqing, China	Preclinical	Mouse model of multidrug-resistant Gram-negative bacterial pneumonia Intravenous administration	PMB (1.0 mg/kg, twice daily)	Significant reduction in bacterial load, oxidative stress and pro-inflammatory cytokines.	[106]
*S. aureus* infection	2014; New York, USA	Preclinical	Mouse skin colonization model (*S. aureus* RN4220, kanamycin-resistant, and USA300) Topical administration	Concentrated phagemid lysate (2 × 10^8^ TU, single topical administration)	Sequence-specific killing of targeted *S. aureus* strains (e.g., RNK, USA300) in vitro and in mouse skin.	[107]
Multidrug-resistant *A. baumannii* infection	2016; Belgium	Preclinical	A panel of 33 *A. baumannii* strains (including multidrug-resistant isolates) and their persister cells In vitro treatment	N/A (in vitro)	Art-175 effectively killed multidrug-resistant *A. baumannii*, including stationary-phase cells and persisters and no resistance development after 20 serial passages.	[108]

N/A indicates that the condition was not applicable for the given example.

**Table 2:** Summary of engineered phage-based therapeutic interventions in non-infectious diseases.

Disease	Study Year(s) and Location	Status	Population and Treatment Method	Dose and Frequency	Outcome	References
Ulcerative Colitis (UC) and Diet-Induced Obesity (DIO)	2023–2025; Blacksburg, VA, USA	Preclinical	Mice colonized with *E. coli* K-12 Oral administration	One phage (10^7^ PFU, single oral administration)	T4::*serpin* phage reduced neutrophil elastase activity and improved weight recovery in DSS-induced colitis mice; T4::clpB phage reduced weight gain and food intake in DIO mice.	[109]
Inflammatory Bowel Disease (IBD)	2025; Nanjing, China	Preclinical	Specific pathogen-free (SPF) mice with DSS-induced colitis and colonized with ST11 KL64 CR-hvKP21 strain Oral administration	One phage (10^10^ PFU, oral gavage once daily for 5 consecutive days)	Significantly reduced CR-hvKP21 bacterial load in feces and mucosa, and enhanced intestinal barrier integrity.	[110]
Various diseases associated with gut microbiota	2023–2024; Paris, France	Preclinical	SPF female BALB/c mice colonized with engineered or pathogenic strains of *Escherichia coli* Oral administration	Phage cocktail (range from 4 × 10^8^ to 4 × 10^11^ transducing units, single-dose and multiple-dose regimens)	Achieved a median editing efficiency of 93% in the target *E. coli* population in the mouse gut with a single dose for a model gene (*bla*) and edited bacteria were stably maintained in the gut for at least 42 days.	[57]
Colorectal Cancer (CRC)	2019; Wuhan, China	Preclinical	Mouse models of colorectal cancer Oral and intravenous administration	One phage (10^11^ PFU, administered once per week)	Effectively reduced intratumoural *F. nucleatum* and increased beneficial butyrate-producing bacteria and butyrate levels.	[118]
Colorectal Cancer (CRC)	2020; Wuhan, China	Preclinical	Mouse models (orthotopic and subcutaneous colorectal tumor models) Intravenous administration	One phage (10^12^ PFU, administered every 3 days)	M13@Ag specifically targeted and eliminated *Fusobacterium nucleatum* in the gut.	[119]
Breast Cancer (BCa)	2020; Oklahoma, USA	Preclinical	Mouse models with orthotopic MCF-7 breast tumors Intravenous administration	One phage (10^12^ PFU, administered on day 10 and day 17)	Significant inhibition of tumor angiogenesis and reduction in microvessel density and tumor volume reduction.	[120]

## 5. Current Limitations and Future Prospects of Engineered Phage Therapy

Despite their remarkable diversity in species and function, which makes phages a highly flexible and scalable biotechnology platform, research in this field remains at an early stage. Although phage therapy has regained attention over the past two decades, public perception and regulatory policies vary significantly across countries, partly because phages are living biologicals. In the USA and the European Union, phages are classified as medicinal products and are subject to strict regulatory constraints, including compliance with Good Manufacturing Practice (GMP) standards [121]. Manufacturing GMP-certified medicinal products is costly and time-consuming, which poses significant challenges to initiating phage-based clinical trials [122]. Nonetheless, progress continues. In 2025, the European Medicines Agency released a draft guideline on quality standards for phage therapy products, outlining requirements for production, quality control, and regulation [123]. In 2016, U.S. physicians conducted the country’s first recent phage therapy under an emergency Investigational New Drug (eIND) application to treat a patient with severe drug-resistant *A. baumannii* infection [31]. Currently, phage therapy in the U.S. is primarily pursued through the IND pathway, which requires FDA authorization, Institutional Review Board approval, and informed patient consent. Similarly, China published the Expert consensus on management of clinical application of phage therapy in 2024, specifying requirements for healthcare institutions, personnel, and technical standards.

### 5.1. Current Challenges

Firstly, at the fundamental biological level, the functions of many phage genes and proteins remain incompletely characterized, which impedes precise and efficient engineering. Concurrently, the inherently limited genomic capacity of phages presents a natural constraint on their utility as delivery vectors. Additionally, any genetic modification risks unintentionally compromising critical innate properties essential for phage survival, such as host recognition, replication efficiency, and environmental stability, making it difficult to maintain a functional balance [124,125,126]. Secondly, regarding clinical translation, the time-consuming, personalized screening model of traditional phage therapy may delay treatment beyond the optimal therapeutic window for acute infections. Furthermore, the in vivo pharmacokinetics of phages are complex [127]; upon systemic administration, they are susceptible to rapid recognition and clearance by the immune system (e.g., the complement system and the reticuloendothelial system), hindering effective delivery to specific sites (e.g., tissues behind the blood–brain barrier or solid tumors) [34,128]. Moreover, on the manufacturing and regulatory front, ensuring the sterility and batch-to-batch consistency of phage preparations is a major hurdle, given the risk of bacterial or viral contamination during production [129]. There is an urgent need to establish end-to-end standardized protocols—from phage isolation and characterization to large-scale production, purification, and quality control—to meet the rigorous safety and efficacy requirements set by drug regulatory agencies [130]. Additionally, the potential environmental release of engineered phages raises inescapable safety concerns, as their ecological impact via horizontal gene transfer requires stringent assessment [131]. Finally, public awareness and acceptance of phages, particularly genetically modified ones, constitute a critical societal factor influencing their market adoption and clinical translation. The longstanding negative perception of “viruses” and general apprehensions surrounding genetically engineered organisms must be addressed through scientific communication and transparent dialogue.

### 5.2. Economic Feasibility and Necessity of Engineered Phage Development

Beyond conventional monophage therapy, expanded strategies—including phage cocktails, phage-antibiotic combinations, and localized delivery—have mitigated key limitations of phage therapy. Phage cocktails expand host spectrum through synergistic targeting of diverse bacterial strains [132]; phage-antibiotic combinations exploit dual mechanisms of action to delay resistance development and enhance bacterial clearance [133]; and localized delivery minimizes systemic phage clearance via immune-mediated neutralization while maximizing site-specific bactericidal efficacy [134]. Despite the immediate cost-effectiveness and practicality of these approaches in resource-limited or emergency settings, engineered phage therapies represent an inevitable evolution toward precision medicine. As synthetic biology, high-throughput screening, and AI-driven design mature and become more cost-efficient, the rational engineering of phages will achieve greater standardization and scalability. Critically, engineered phages offer superior therapeutic efficacy, reduced off-target effects, and the potential for sustained bacterial eradication—yielding enhanced cost-effectiveness compared to iterative antibiotic use or complex phage cocktail regimens. Consequently, sustained investment in engineered phage R&D constitutes not merely a rational economic choice, but a strategic imperative for future antimicrobial therapeutics.

### 5.3. Future Prospects and Research Directions

Engineered phages demonstrate considerable potential in combating MDR bacterial infections. However, their translation from laboratory research to broad clinical application and commercialization continues to face systematic challenges. To accelerate this transition, coordinated efforts in the following key areas are urgently required:

First, the establishment of a functional annotation framework for phage genomes and the construction of a modular engineering toolkit represent foundational breakthroughs. There is an urgent need to create an open-source, globally shared functional annotation database for phage genomes, to develop computational models capable of predicting host range and immune evasion capabilities, and to standardize the assembly and functional validation of modular phage chassis. These steps would significantly shorten the development cycle for customized phages.

Second, optimizing the pharmacokinetic (PK)/pharmacodynamic (PD) profiles of engineered phages and innovating delivery systems are core to enhancing in vivo efficacy. Specific approaches include establishing standardized PK/PD animal models and in vitro infection models, developing intelligent delivery systems responsive to pH or enzymes (e.g., microencapsulated oral formulations or nebulized inhalants), and completing the first human Phase I pharmacokinetic study for an engineered phage therapeutic.

Third, clinical validation targeting high-burden multidrug-resistant infections is crucial for building the evidence chain necessary for therapeutic adoption. In response to global public health threats such as *A. baumannii*, *P. aeruginosa* (Gram-negative bacteria), MRSA, and *C. difficile* (Gram-positive bacteria), it is imperative to conduct Phase II/III randomized controlled trials (RCTs) of engineered phages targeting these pathogens. This will provide confirmatory data on efficacy and safety, informing the development of clinical practice guidelines covering patient selection, dosing regimens, and dynamic monitoring.

Finally, the establishment of a regulatory framework and industrialization standards for phage therapy is the institutional guarantee for ensuring scalable application. Key tasks include publishing internationally recognized manufacturing and quality control specifications for engineered phages, refining preclinical safety evaluation systems, and facilitating the first conditional marketing authorization for an engineered phage product in regions such as Europe, the United States, or China.

In summary, the advancement of engineered phage technology constitutes a systemic endeavor. Its successful development and breakthrough hinge upon innovation in core technologies, optimization of clinical protocols, refinement of regulatory frameworks, and establishment of public trust. Only through concerted efforts to overcome these multifaceted challenges can the immense application potential of this powerful biological platform be fully unlocked. We firmly believe that engineered phages, as highly customizable and precise biological tools, will not only provide a transformative solution to the antibiotic resistance crisis but also greatly promote the convergence of synthetic biology, medicine, and environmental science, ushering in a new era of precision phage medicine.

## Data Availability

No new data were created or analyzed in this study.

## Abbreviations

The following abbreviations are used in this manuscript:

AMR	Antimicrobial Resistance
HRDR	Host Range Determinant Region
ACR	Anti-CRISPR
MRSA	Methicillin-Resistant *Staphylococcus aureus*
PEG	Polyethylene Glycol
MDR	Multidrug-Resistant
XDR	Extensively Drug-Resistant
EPEs	Engineered Phage Enzymes
IBD	Inflammatory Bowel Disease
CRC	Colorectal Cancer
NAFLD	Non-Alcoholic Fatty Liver Disease
NE	Neutrophil Elastase
CRKP	Carbapenem-Resistant *Klebsiella pneumoniae*
TME	Tumor Microenvironment
UC	Ulcerative Colitis
DIO	Diet-Induced Obesity
BCa	Breast Cancer
GMP	Good Manufacturing Practice
eIND	emergency Investigational New Drug
PK	Pharmacokinetic
PD	Pharmacodynamic
RCTs	Randomized Controlled Trials
